# Supplementary material for: Fusobacterium nucleatum reduces METTL3-mediated m6A modification and contributes to colorectal cancer metastasis
Source: Nat Commun. 2022 Mar 10;13:1248. doi: 10.1038/s41467-022-28913-5 (PMC8913623; doi:10.1038/s41467-022-28913-5)
Supplement: Supplementary file 1 — Supplementary Information [file 41467_2022_28913_MOESM1_ESM.pdf]

Supplementary Information

Fusobacterium nucleatum Reduces METTL3-mediated m^6A Modification and Contributes to Colorectal Cancer Metastasis

Shujie Chen, Lu Zhang, Mengjie Li, Ying Zhang, Meng Sun, Lingfang Wang, Jiebo Lin, Yun Cui, Qian Chen, Chenqi Jin, Xiang Li, Boya Wang, Hao Chen, Tianhua Zhou, Liangjing Wang, Chih-Hung Hsu, Wei Zhuo

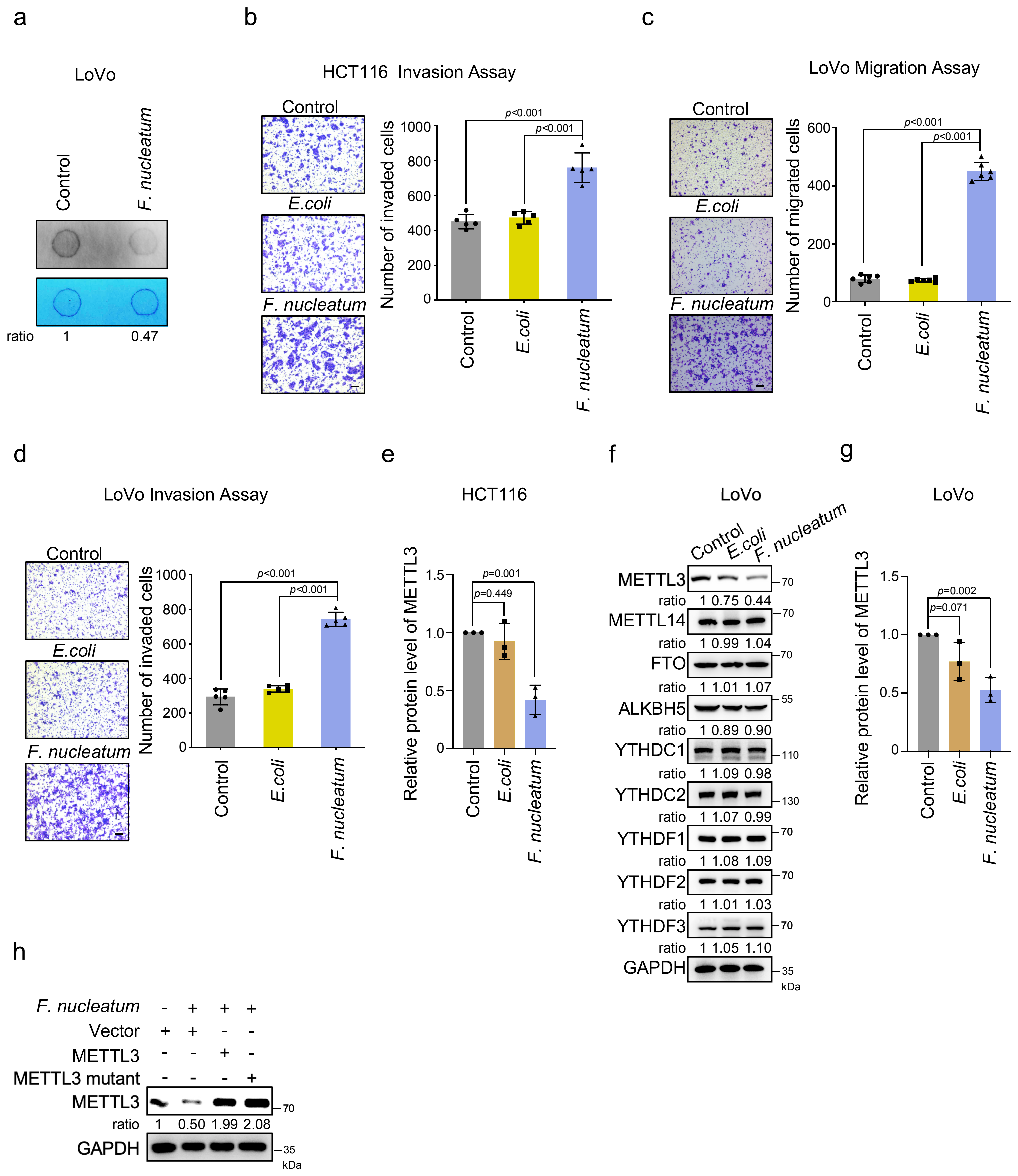
a
LoVo
Control
F. nucleatum
ratio 1 0.47
b
HCT116 Invasion Assay
Control
E.coli
F. nucleatum
Number of invaded cells
p<0.001
p<0.001
c
LoVo Migration Assay
Number of migrated cells
d
LoVo Invasion Assay
e
HCT116
Relative protein level of METTL3
p=0.001
p=0.449
f
LoVo
METTL3
ratio 1 0.75 0.44
METTL14
ratio 1 0.99 1.04
FTO
ratio 1 1.01 1.07
ALKBH5
ratio 1 0.89 0.90
YTHDC1
ratio 1 1.09 0.98
YTHDC2
ratio 1 1.07 0.99
YTHDF1
ratio 1 1.08 1.09
YTHDF2
ratio 1 1.01 1.03
YTHDF3
ratio 1 1.05 1.10
GAPDH
kDa
g
LoVo
p=0.002
p=0.071
h
F. nucleatum - + + +
Vector + + - -
METTL3 - - + -
METTL3 mutant - - - +
METTL3
ratio 1 0.50 1.99 2.08
GAPDH
kDa

Supplementary Fig. 1 *F. nucleatum* downregulates m^{6}A levels and METTL3 expression in CRC cells.

a, mRNA dot blot analysis was performed to determine the m^{6}A levels of LoVo cells co-cultured with or without *F. nucleatum*.

b, HCT116 cells were pretreated with *F. nucleatum* , *E.coli* DH5α or PBS control for 2 h and subjected to invasion assay (Left). The invaded cells were quantified by counting in five fields (Right). Scale bar, 100 μm.

c, LoVo cells were pretreated with *F. nucleatum*, *E.coli* DH5 or PBS control for 2 h and subjected to transwell assay (Left). The migrated cells were quantified by counting in six fields (Right). Scale bar, 100 μm.

d, LoVo cells were pretreated with *F. nucleatum* , *E.coli* DH5α or PBS control for 2 h and subjected to invasion assay (Left). The invaded cells were quantified by counting in five fields (Right). Scale bar, 100 μm.

e, The statistical analysis of METTL3 protein levels from three independent experiments of HCT116 cells treated with *F. nucleatum* , *E.coli* DH5α or PBS control.

f, Western blot was performed to determine the m^{6}A modification-associated protein levels in LoVo cells treated with *F. nucleatum*, *E.coli* DH5 or PBS control.

g, The statistical analysis of METTL3 protein levels from three independent experiments of LoVo cells treated with *F. nucleatum* , *E.coli* DH5α or PBS control.

h, Western blot was performed to detect the expression of METTL3 in HCT116 cells transfected with siRNA targeting METTL3 or control siRNAs.

The methylene blue staining was used as a loading control in mRNA dot blot assay. Data are from one representative of three independent experiments (**a**, **f**, **h**). Data are shown as mean ± SD. *P*-values was shown. Two-tailed Student's t test (**b, c, d, e, g**).

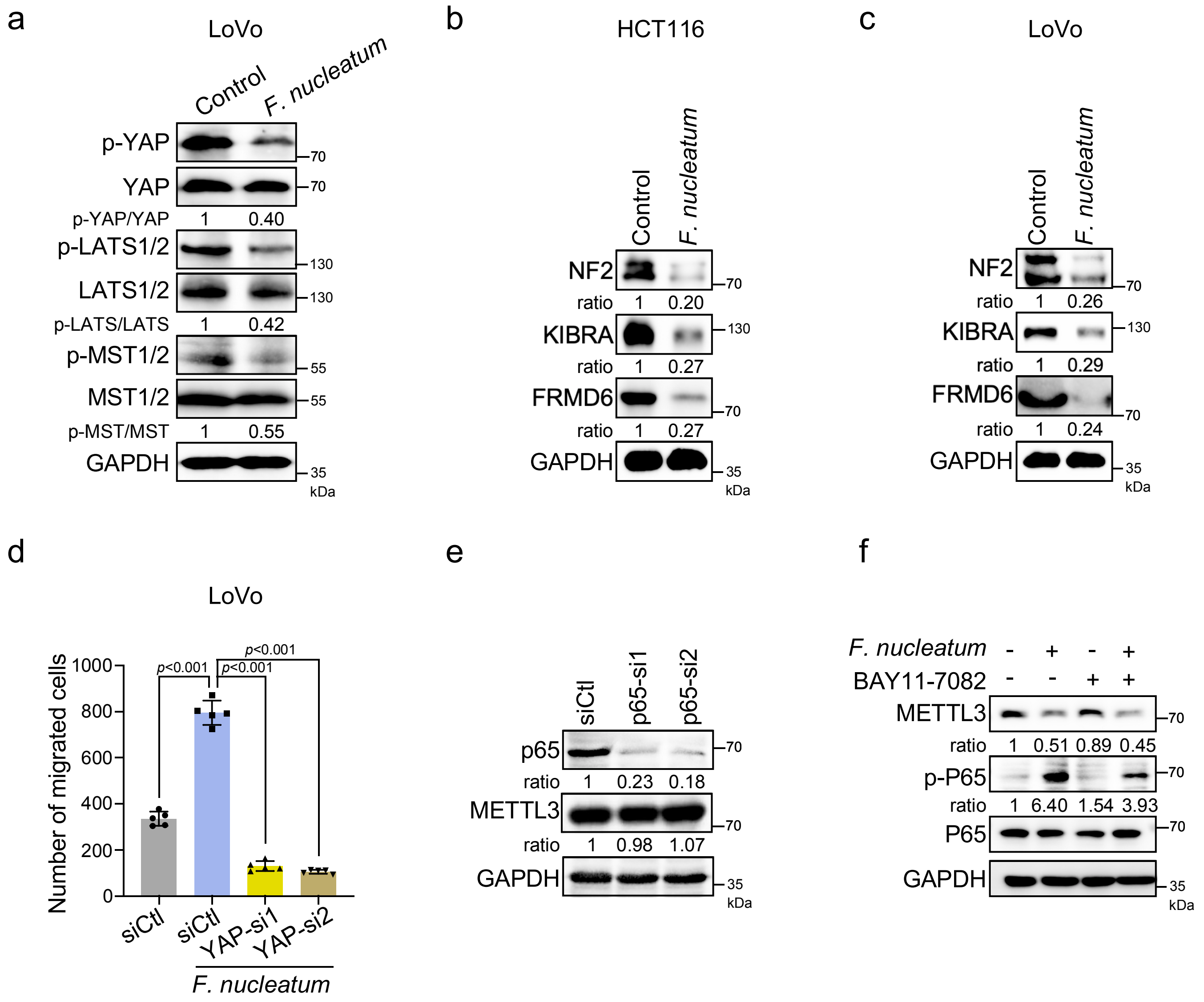
a
LoVo
Control
F. nucleatum
p-YAP
YAP
p-YAP/YAP 1 0.40
p-LATS1/2
LATS1/2
p-LATS/LATS 1 0.42
p-MST1/2
MST1/2
p-MST/MST 1 0.55
GAPDH
70
70
130
130
55
55
35
kDa
b
HCT116
Control
F. nucleatum
NF2
ratio 1 0.20
KIBRA
ratio 1 0.27
FRMD6
ratio 1 0.27
GAPDH
c
LoVo
Control
F. nucleatum
NF2
ratio 1 0.26
KIBRA
ratio 1 0.29
FRMD6
ratio 1 0.24
GAPDH
d
LoVo
Number of migrated cells
p<0.001
p<0.001
p<0.001
siCtl
siCtl
YAP-si1
YAP-si2
F. nucleatum
e
siCtl
p65-si1
p65-si2
p65
ratio 1 0.23 0.18
METTL3
ratio 1 0.98 1.07
GAPDH
f
F. nucleatum - + - +
BAY11-7082 - - + +
METTL3
ratio 1 0.51 0.89 0.45
p-P65
ratio 1 6.40 1.54 3.93
P65
GAPDH

Supplementary Fig. 2 *F. nucleatum* activates YAP signaling in CRC cells.

a, Western blot was performed to detect the levels of YAP and phospho-YAP, LATS1/2 and phospho-LATS1/2, MST1/2 and phospho-MST1/2 in LoVo cells treated with *F. nucleatum* or PBS control.

b, **c**, Western blot was performed to detect the expression levels of NF2, KIBRA and FRMD6 in HCT116 (**b**) or LoVo cells (**c**) treated with *F. nucleatum* or PBS control.

d, LoVo cells were transfected with two different siRNAs against YAP or scrambled control siRNAs and treated with *F. nucleatum* or PBS control. Transwell migration assay was performed. The migrated cells were quantified by counting in five fields.

e, HCT116 cells transfected with two different siRNAs against p65 or scrambled control siRNAs were subjected to western blot analysis of METTL3.

f, After treating with *F. nucleatum* or PBS control, HCT116 cells were administrated with BAY11-7082 and subjected to western blot analysis for METTL3, p-P65 and P65. Data are from one representative of three independent experiments (**a**-**c**, **e**-**f**). Data are shown as mean ± SD. *P*-values was shown. Two-tailed Student's t test (**d**)

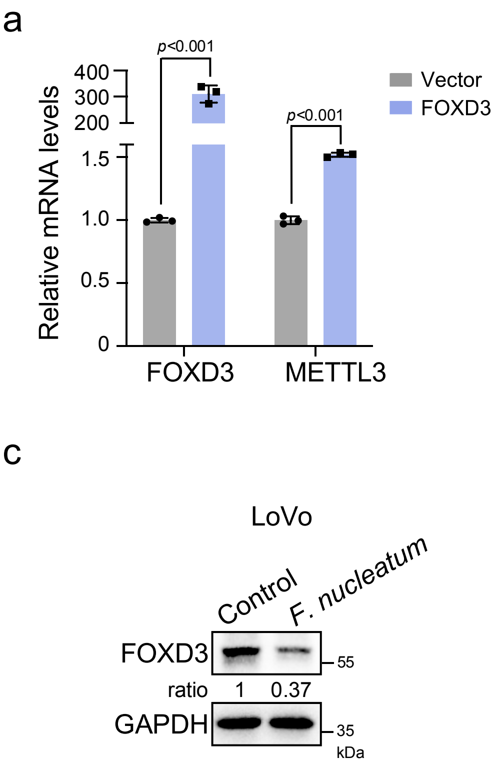

a
p<0.001
p<0.001
Relative mRNA levels
400
300
200
1.5
1.0
0.5
0
FOXD3
METTL3
Vector
FOXD3
b
p<0.001
p<0.001
Relative mRNA levels
1.5
1.0
0.5
0
FOXD3
METTL3
Control
FOXD3-KD
c
LoVo
Control
F. nucleatum
FOXD3
55
ratio 1 0.37
GAPDH
35
kDa

Supplementary Fig. 3 YAP and FOXD3 as the upstream regulators of METTL3 participate in its transcriptional regulation and the levels of FOXD3 were decreased in *F. nucleatum*-treated LoVo cells.

a, Quantitative RT-PCR was performed in HCT116 cells to detect the expression of *METTL3* mRNA after transfection with FOXD3 plasmid or control vector.

b, Quantitative RT-PCR was performed in HCT116 cells to detect the expression of *METTL3* mRNA after transfection with siRNA targeting FOXD3.

c, Western blot analysis of FOXD3 expression in LoVo cells treated with *F. nucleatum* or PBS control.

Data are from one representative of three independent experiments (**c**). Data are shown as mean ± SD. *P*-values was shown. Two-tailed Student's t test (**a, b**)

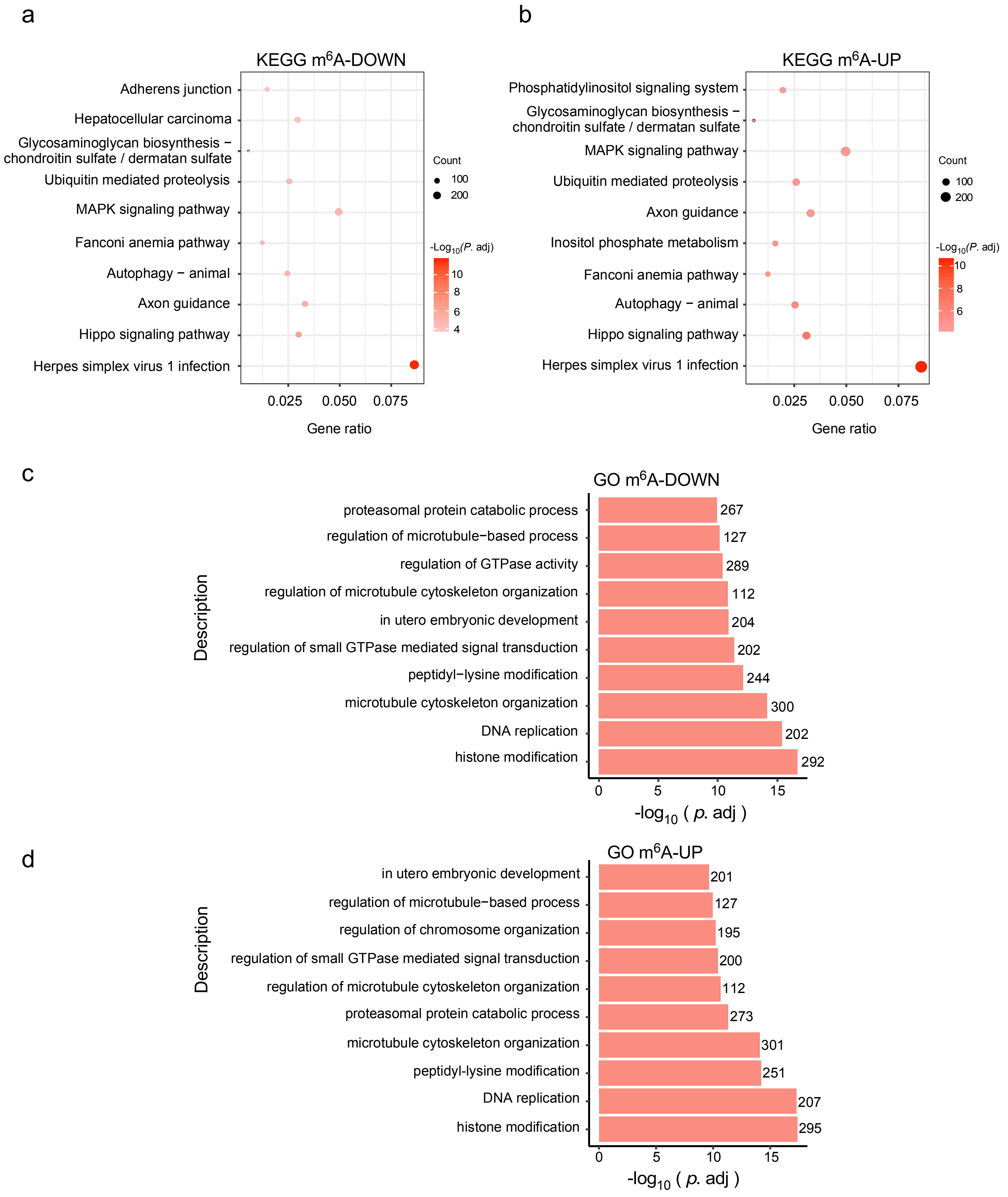
a
KEGG m6A-DOWN
Adherens junction
Hepatocellular carcinoma
Glycosaminoglycan biosynthesis − chondroitin sulfate / dermatan sulfate
Ubiquitin mediated proteolysis
MAPK signaling pathway
Fanconi anemia pathway
Autophagy − animal
Axon guidance
Hippo signaling pathway
Herpes simplex virus 1 infection
0.025 0.050 0.075
Gene ratio
Count
100
200
-Log10(P. adj)
10
8
6
4
b
KEGG m6A-UP
Phosphatidylinositol signaling system
Glycosaminoglycan biosynthesis − chondroitin sulfate / dermatan sulfate
MAPK signaling pathway
Ubiquitin mediated proteolysis
Axon guidance
Inositol phosphate metabolism
Fanconi anemia pathway
Autophagy − animal
Hippo signaling pathway
Herpes simplex virus 1 infection
0.025 0.050 0.075
Gene ratio
Count
100
200
-Log10(P. adj)
10
8
6
c
GO m6A-DOWN
Description
proteasomal protein catabolic process 267
regulation of microtubule−based process 127
regulation of GTPase activity 289
regulation of microtubule cytoskeleton organization 112
in utero embryonic development 204
regulation of small GTPase mediated signal transduction 202
peptidyl−lysine modification 244
microtubule cytoskeleton organization 300
DNA replication 202
histone modification 292
0 5 10 15
-log10 (p. adj)
d
GO m6A-UP
Description
in utero embryonic development 201
regulation of microtubule−based process 127
regulation of chromosome organization 195
regulation of small GTPase mediated signal transduction 200
regulation of microtubule cytoskeleton organization 112
proteasomal protein catabolic process 273
microtubule cytoskeleton organization 301
peptidyl-lysine modification 251
DNA replication 207
histone modification 295
0 5 10 15
-log10 (p. adj)

Supplementary Fig. 4 Enrichment analysis of m^{6}A-regulated genes in CRC cells treated with *F. nucleatum*.

a, KEGG pathway analysis of a total number of 2004 genes with significant differential m^{6}A-down peaks in *F. nucleatum*-treated cells compared with untreated cells.

b, KEGG pathway analysis of a total number of 1585 genes with significant differential m^{6}A-up peaks in *F. nucleatum*-treated cells compared with untreated cells.

c, Gene Ontology enrichment analysis of a total number of 2004 genes with differential m^{6}A-down peaks in *F. nucleatum*-treated cells compared with untreated cells.

d, Gene Ontology enrichment analysis of a total number of 1585 genes with differential m^{6}A-up peaks in *F. nucleatum*-treated cells compared with untreated cells. -log10 (*p*.adj) of the 10 most enriched pathways or gene functions related to biological process are displayed.

KEGG and GO were analyzed using hypergeometric test adjusted with Benjamini-Hochberg method (*P*. adj).

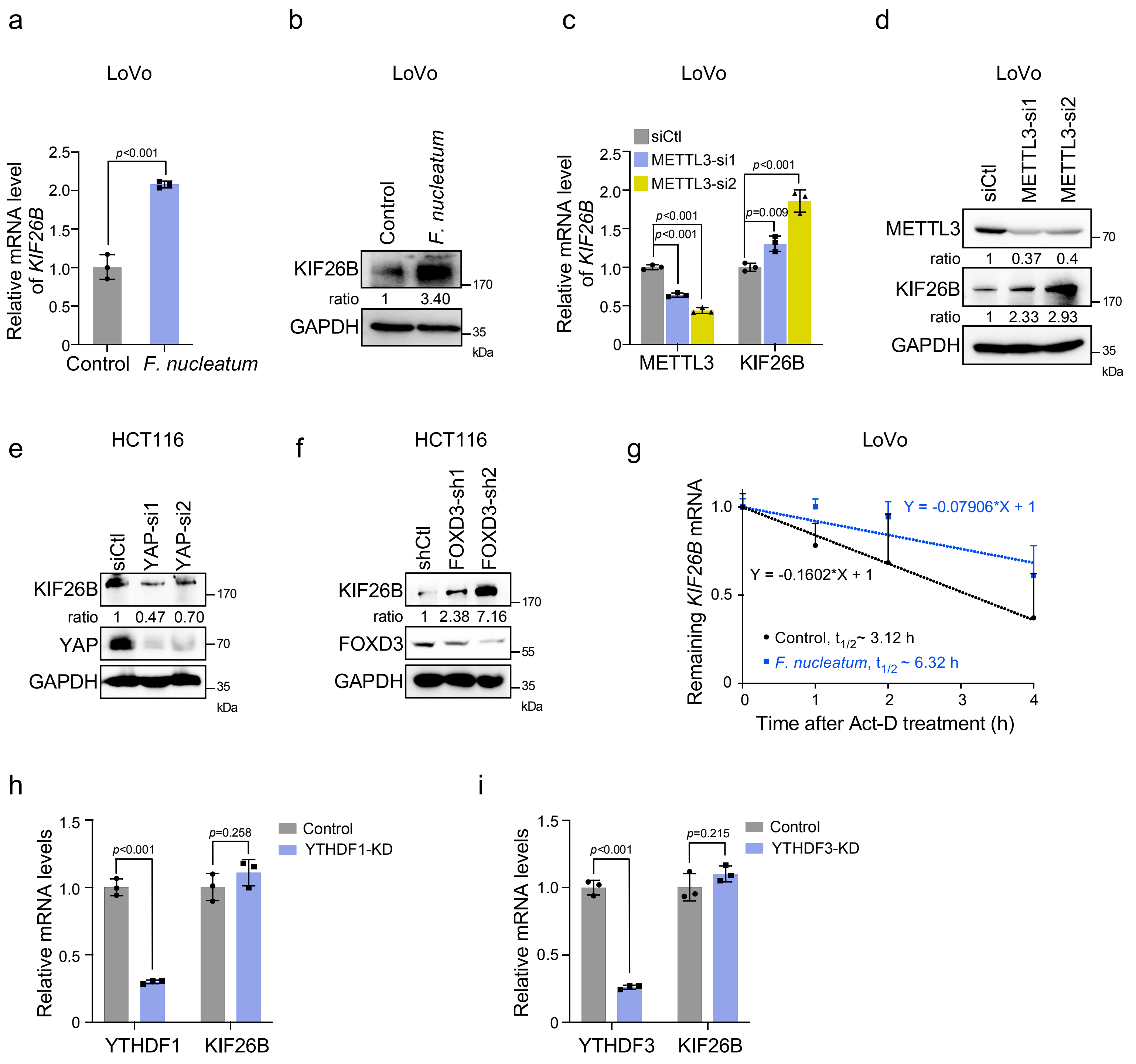

a
LoVo
Relative mRNA level of KIF26B
p<0.001
Control
F. nucleatum
b
LoVo
Control
F. nucleatum
KIF26B
ratio 1 3.40
GAPDH
170
35
kDa
c
LoVo
siCtl
METTL3-si1
METTL3-si2
Relative mRNA level of KIF26B
p<0.001
p<0.001
p<0.001
p=0.009
METTL3
KIF26B
d
LoVo
siCtl
METTL3-si1
METTL3-si2
METTL3
ratio 1 0.37 0.4
KIF26B
ratio 1 2.33 2.93
GAPDH
70
170
35
kDa
e
HCT116
siCtl
YAP-si1
YAP-si2
KIF26B
ratio 1 0.47 0.70
YAP
GAPDH
170
70
35
kDa
f
HCT116
shCtl
FOXD3-sh1
FOXD3-sh2
KIF26B
ratio 1 2.38 7.16
FOXD3
GAPDH
170
55
35
kDa
g
LoVo
Remaining KIF26B mRNA
Y = -0.07906*X + 1
Y = -0.1602*X + 1
Control, t1/2~ 3.12 h
F. nucleatum, t1/2 ~ 6.32 h
Time after Act-D treatment (h)
h
Relative mRNA levels
p<0.001
p=0.258
Control
YTHDF1-KD
YTHDF1
KIF26B
i
Relative mRNA levels
p<0.001
p=0.215
Control
YTHDF3-KD
YTHDF3
KIF26B

Supplementary Fig. 5 KIF26B is a downstream target of METTL3.

a, **b**, LoVo cells treated with *F. nucleatum* or PBS control were subjected to quantitative RT-PCR analysis (**a**) and western blot analysis (**b**) of KIF26B expression.

c, **d**, LoVo cells transfected with two different siRNAs targeting METTL3 or scrambled control siRNAs were subjected to quantitative RT-PCR analysis (**c**) and western blot analysis (**d**) of KIF26B expression.

e, HCT116 cells with the indicated treatment were subjected to western blot analysis of KIF26B.

f, HCT116 cells transfected with shRNAs targeting FOXD3 or control shRNA were subjected to western blot analysis of KIF26B

g, LoVo cells were pretreated with *F. nucleatum* or PBS for 2 h. The remaining levels of *KIF26B* mRNAs were analyzed by quantitative RT-PCR at the indicated time points after actinomycin D treatment. Data from one representative of three independent experiments.

h, **i**, Quantitative RT-PCR was performed in HCT116 cells to detect the expression of *KIF26B* mRNA after transfection with siRNA targeting YTHDF1 (**h**) or YTHDF3 (**i**).

Data are from one representative of three independent experiments (**b, d**-**f**). Data are shown as mean ± SD. *P*-values was shown. Two-tailed Student's t test (**a, c, h, i**)

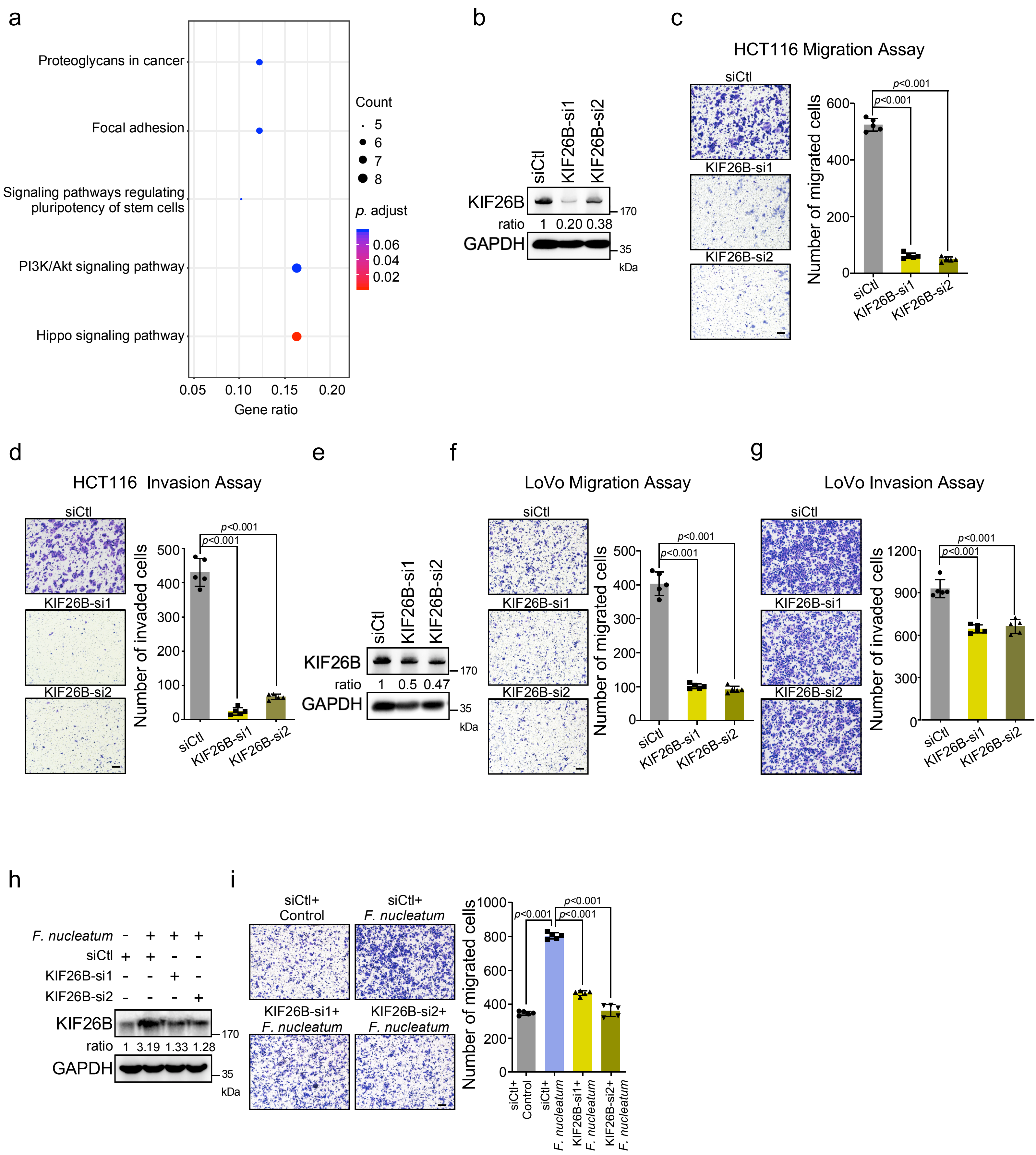

a
Proteoglycans in cancer
Focal adhesion
Signaling pathways regulating pluripotency of stem cells
PI3K/Akt signaling pathway
Hippo signaling pathway
Count
5
6
7
8
p. adjust
0.06
0.04
0.02
0.05
0.10
0.15
0.20
Gene ratio
b
siCtl
KIF26B-si1
KIF26B-si2
KIF26B
ratio 1 0.20 0.38
GAPDH
170
35
kDa
c
HCT116 Migration Assay
siCtl
KIF26B-si1
KIF26B-si2
Number of migrated cells
p<0.001
p<0.001
d
HCT116 Invasion Assay
Number of invaded cells
p<0.001
p<0.001
e
ratio 1 0.5 0.47
f
LoVo Migration Assay
g
LoVo Invasion Assay
h
F. nucleatum - + + +
siCtl + + - -
KIF26B-si1 - - + -
KIF26B-si2 - - - +
ratio 1 3.19 1.33 1.28
i
siCtl+ Control
siCtl+ F. nucleatum
KIF26B-si1+ F. nucleatum
KIF26B-si2+ F. nucleatum

Supplementary Fig. 6 *F. nucleatum* promotes CRC cell migration by upregulating KIF26B in *vitro*.

a, KEGG pathway analysis of the 123 downregulated genes from the RNA-seq data of the KIF26B-knockdown HCT116 cells and control cells. *P*. adjust, hypergeometric test with Benjamini-Hochberg adjusted.

b-d, HCT116 cells were transfected with two siRNAs targeting KIF26B or control siRNAs and subjected to western blot analysis (**b**), transwell migration analysis (**c**) and invasion analysis (**d**). Migrated cells were quantified by counting in five fields. Scale bar, 100 μm.

e-g, LoVo cells were transfected with two siRNAs targeting KIF26B or control siRNAs and subjected to western blot analysis (**e**), transwell migration analysis (**e**) and invasion analysis (**g**). Migrated cells were quantified by counting in five fields. Scale bar, 100 μm.

h, **i**, LoVo cells transfected with two siRNAs targeting KIF26B or control siRNAs were treated with *F. nucleatum* for 2 h. Western blot analysis (**h**) and transwell migration analysis (**i**) were performed. Scale bar, 100 μm.

Data are from one representative of three independent experiments (**b**, **e**, **h**). Data are shown as mean ± SD. *P*-values was shown. Two-tailed Student's t test (**c, d, f, g, i**)

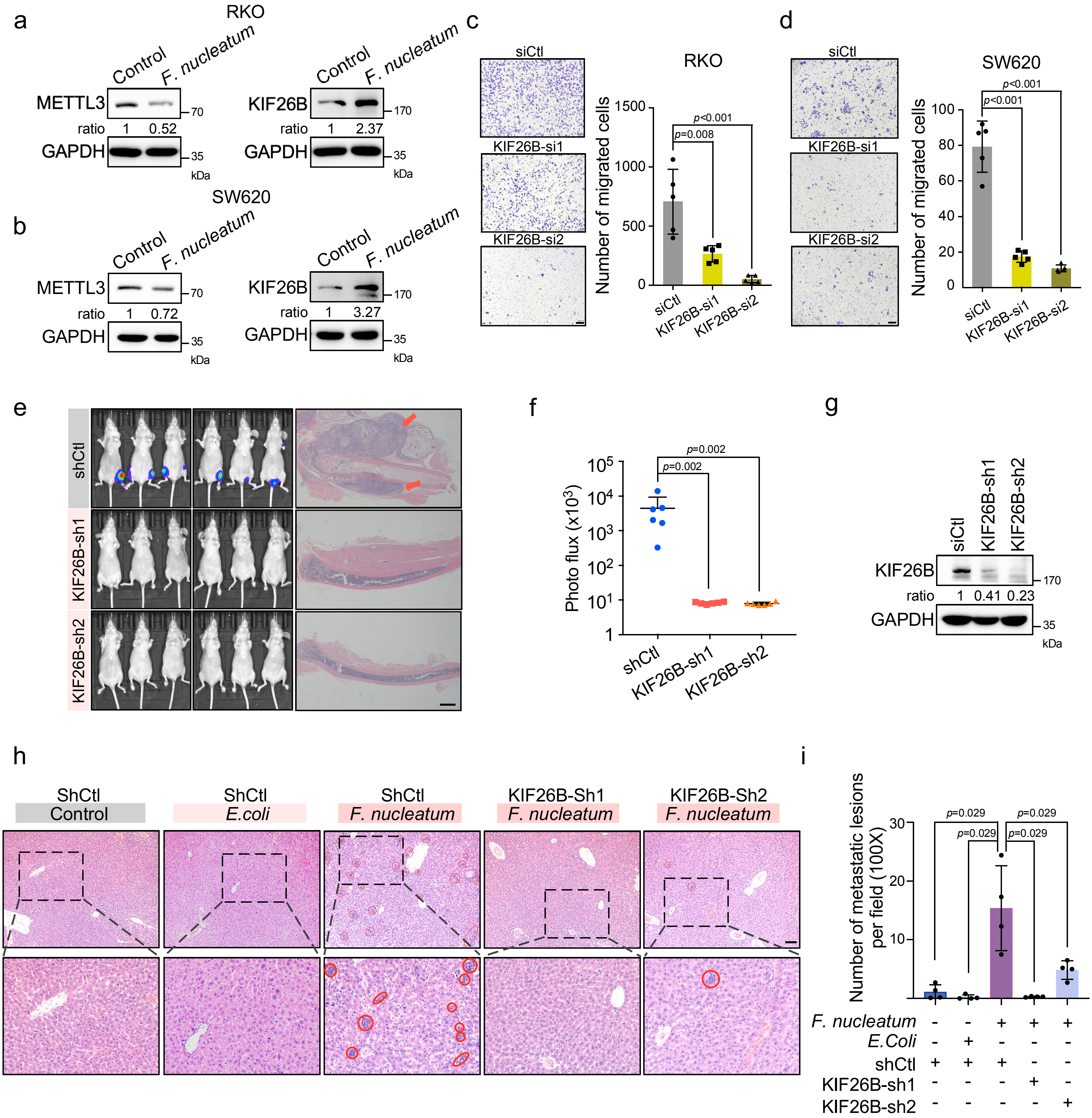

a
RKO
Control
F. nucleatum
METTL3
ratio 1 0.52
GAPDH
KIF26B
ratio 1 2.37
70
35
170
kDa
b
SW620
METTL3
ratio 1 0.72
GAPDH
KIF26B
ratio 1 3.27
c
siCtl
KIF26B-si1
KIF26B-si2
RKO
Number of migrated cells
p=0.008
p<0.001
d
SW620
p<0.001
p<0.001
e
shCtl
KIF26B-sh1
KIF26B-sh2
f
Photo flux (x10³)
p=0.002
p=0.002
g
siCtl
KIF26B-sh1
KIF26B-sh2
ratio 1 0.41 0.23
h
ShCtl
Control
E.coli
KIF26B-Sh1
KIF26B-Sh2
i
Number of metastatic lesions per field (100X)
p=0.029
F. nucleatum
E.Coli
shCtl

Supplementary Fig. 7 ***F. nucleatum*** **accelerates CRC aggressiveness and metastasis by upregulating KIF26B.**

a, **b**, RKO cells (**a**) or SW620 cells (**b**) treated with *F. nucleatum* or PBS control were subjected to western blot analysis of METTL3 (Left) and KIF26B (Right) expression.

c, **d**, RKO cells (**c**) or SW620 cells (**d**) were transfected with two siRNAs targeting KIF26B or control siRNAs and subjected to transwell migration analysis (Left). Migrated cells were quantified by counting in five fields (Right). Scale bar, 100 μm.

e, **f**, KIF26B-knockdown luciferase-labeled RKO cells or the corresponding control cells were intravenously injected into nude mice. Representative bioluminescence images and H&E stained bone sections of the mice are shown (Scale bar, 10 μm) (**e**). BLI monitored the bone metastases formed from injected cells (**f**). (n = 6)

g, Western blot analysis of KIF26B expression in HCT116 cells infected with two different shRNAs targeting KIF26B or control shRNA.

h, HCT116 cells were stably infected with lentivirus-based KIF26B shRNAs or control shRNAs. The indicated cells were orthotopically injected into NOD SCID mice to develop liver metastasis. H&E stained liver sections of the mice are shown, Scale bar, 100 μm.

i, Liver micro-metastases per mice were quantified by counting in ten fields. (n = 4)

Data are from one representative of three independent experiments (**a**, **b**, **g**). Data are shown as mean ± SD. *P*-values was shown. Two-tailed Student's t test (**c, d**), Two-tailed Mann-Whitney test (**f**, **i**).

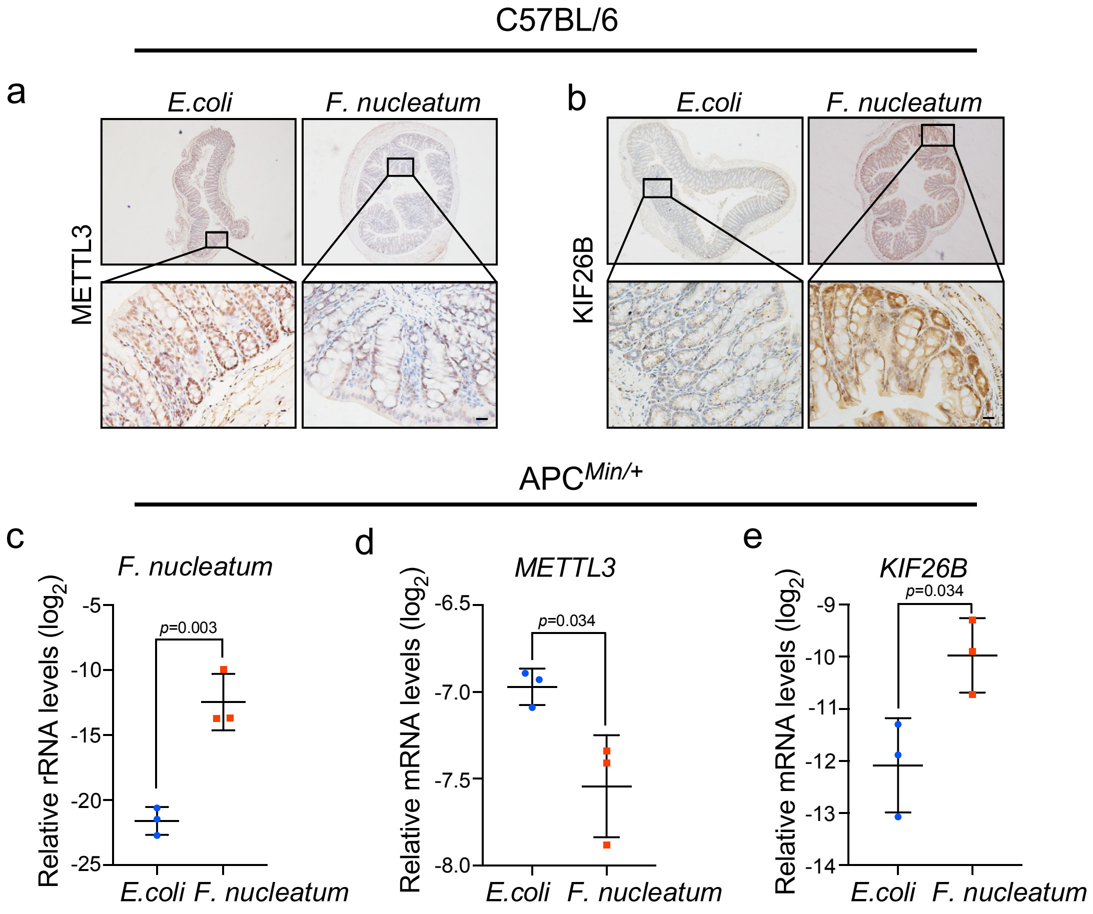
C57BL/6
a
E.coli
F. nucleatum
METTL3
b
E.coli
F. nucleatum
KIF26B
APC$^{Min/+}$
c
F. nucleatum
Relative rRNA levels (log$_2$)
p=0.003
-5
-10
-15
-20
-25
E.coli
F. nucleatum
d
METTL3
Relative mRNA levels (log$_2$)
p=0.034
-6.5
-7.0
-7.5
-8.0
E.coli
F. nucleatum
e
KIF26B
Relative mRNA levels (log$_2$)
p=0.034
-9
-10
-11
-12
-13
-14
E.coli
F. nucleatum

Supplementary Fig. 8 *F. nucleatum* is correlated with *METTL3* and *KIF26B* expressions in animal model.

a, **b,** Representative immunohistochemistry images of METTL3 (**a**) and KIF26B (**b**) proteins in colorectum tissues from mice with the indicated treatment. Scale bar, 20 μm.

c, Quantitative RT-PCR analysis of *F. nucleatum* in stool from the indicated $APC^{Min/+}$ mice (n = 3). Data are presented as $\log_2$ value of *F. nucleatum 16S* normalized to universal *Eubacteria 16S*.

d, e, Quantitative RT-PCR analysis of *METTL3* (**d**) and *KIF26B* (**e**) mRNA expression in colorectum tissues from the indicated $APC^{Min/+}$ mice (n = 3). Data are presented as $\log_2$ value normalized to *GAPDH.*

Data are shown as mean ± SD. *P*-values was shown. Two-tailed Mann-Whitney test (**c**-**e**).

Supplementary Table 1. Primers for qRT-PCR

Primer Names	Sequences (5'-3')
METTL3 Forward	CAAGCTGCACTTCAGACGAA
METTL3 Reverse	GCTTGGCGTGTGGTCTTT
FOXD3 Forward	GCAACTACTGGACCCTGGAC
FOXD3 Reverse	CTGTAAGCGCCGAAGCTCT
c-Myc Forward	AATGAAAAGGCCCCCAAGGTAGTTATCC
c-Myc Reverse	GTCGTTTCCGCAACAAGTCCTCTTC
CD44 Forward	CTGCCGCTTTGCAGGTGTA
CD44 Reverse	CATTGTGGGCAAGGTGCTATT
AXIN2 Forward	TGTCTTAAAGGTCTTGAGGGTTGAC
AXIN2 Reverse	CAACAGATCATCCCATCCAACA
HES-1 Forward	CCTGTCATCCCCGTCTACAC
HES-1 Reverse	CACATGGAGTCCGCCGTAA
HEY1 Forward	GAAGTTGCGCGTTATCTGAGC
HEY1 Reverse	ATGCGAAACCAGTCGAACTCG
HEY2 Forward	CCTAACAGAAGTTGCGCGGTA
HEY2 Reverse	GAGGCGACAAGGGGTTGAC
CLN3 Forward	ATTCCGAGGGGGAGGAGACC
CLN3 Reverse	AGGGAACAATGTACCACAGCAG
HIF-1α Forward	TCCTGAGGAAGAACTAAATCCAAAG
HIF-1α Reverse	GGCTGCTGTAATAATGTTCCAATTC
TNF-α Forward	GAGGCCAAGCCCTGGTATG
TNF-α Reverse	CGGGCCGATTGATCTCAGC
IL-1 Forward	TTCGACACATGGGATAACGAGG
IL-1 Reverse	TTTTTGCTGTGAGTCCCGGAG
IL-8 Forward	ACTGAGAGTGATTGAGAGTGGAC
IL-8 Reverse	AACCCTCTGCACCCAGTTTTC
ANKRD1 Forward	AGTAGAGGAACTGGTCACTGG
ANKRD1 Reverse	TGGGCTAGAAGTGTCTTCAGAT
THBS1 Forward	CCTGACCGTCCAAGGAAAGC
THBS1 Reverse	CCTTTGCGATGCGGAGTCT
CYR61 Forward	GCATTCCTCTGTGTCCCCAA

CYR61 Reverse	CATTCCAAAAACAGGGAGCCG
CTGF Forward	ACCGACTGGAAGACACGTTTG
CTGF Reverse	CCAGGTCAGCTTCGCAAGG
ETS1 Forward	ACCGTGCTGACCTCAATAAGG
ETS1 Reverse	CCCCGCTGTCTTGTGGATG
KIF26B Forward	GCTGGGAATAAAGAGAGGCTTG
KIF26B Reverse	ACTCCTCGTATGCTTTCCGGT
YTHDF1 Forward	ATACCTCACCACCTACGGACA
YTHDF1 Reverse	GTGCTGATAGATGTTGTTCCCC
YTHDF2 Forward	CCTTAGGTGGAGCCATGATTG
YTHDF2 Reverse	TCTGTGCTACCCAACTTCAGT
YTHDF3 Forward	TCAGAGTAACAGCTATCCACCA
YTHDF3 Reverse	GGTTGTCAGATATGGCATAGGCT
GAPDH Forward	GGAGCGAGATCCCTCCAAAAT
GAPDH Reverse	GGCTGTTGTCATACTTCTCATGG
m*KIF26B* Forward	TCGGTAGCCGGAAATAAAGAGA
m*KIF26B* Reverse	CGACTCCTCGTAAGCCTTGC
m*ACTIN* Forward	AGCCATGTACGTAGCCATCC
m*ACTIN* Reverse	CTCTCAGCTGTGGTGGTGAA
universal Eubacteria 16s Forward	CGGCAACGAGCGCAACCC
universal Eubacteria 16s Reverse	CCATTGTAGCACGTGTGTAGCC
PTG Forward	ATCCCCAAAGCACCTGGTTT
PTG Reverse	AGAGGCCAAGATAGTCCTGGTAA
F. nucleatum Forward	CGGGTGAGTAACG CGTAAAG
F. nucleatum Reverse	ACATTGTGCCACG GACATCTTG
FOXD3-ChIP-1- Forward	TGGGTCATTAAACTTGGAGT
FOXD3-ChIP-1- Reverse	TTCACAGCATGAGGTAGCAT
FOXD3-ChIP-2- Forward	AGGAGCCATGCCAGTCAAAC
FOXD3-ChIP-2- Reverse	TTAATGGAGCTCCCTGAATG
FOXD3-ChIP-3- Forward	TCACCTCAGATTGGGGACCA
FOXD3-ChIP-3- Reverse	CACTAGTTCCTTTTGACAGT
FOXD3-ChIP-unrelate-Forward	TGGAGTGCTGTGGCACAATC
FOXD3-ChIP-unrelate-Reverse	TTAGCTGGGCACGGTGATGG
KIF26B-m^{6}A-RT-qPCR-Forward	GAGGATGAAGGTTGGTGGCA

KIF26B-m^{6}A-RT-qPCR-Reverse	TTCAGTCTCACAGGGCTTGG

Supplementary Table 2. siRNA sequences

Oligonucleotide Names	Sequences (5'-3')
siCtl	UUCUCCGAACGUGUCACGUTT
si*YAP*-1	GACAUCUUCUGGUCAGAGATT
si*YAP*-2	CCGUUUCCCAGACUACCUUTT
si*METTL3*-1	AGGAGCCAGCCAAGAAAUCAATT
si*METTL3*-2	CUGCAAGUAUGUUCACUAUGATT
si*YTHDF1*-1	CCGCGUCUAGUUGUUCAUGAA
si*YTHDF1*-2	CAGGCUGGAGAAUAACGACAA
si*YTHDF2*-1	CCUACCAGAUGCAAUGUUUTT
si*YTHDF2*-2	CCAGCUUUCAGUCCAGCAATT
si*YTHDF3*-1	AUGGAUUAAAUCAGUAUCUAA
si*YTHDF3*-2	UAAGUCAAAGAAGACGUAUUA
si*KIF26B*-1	GCUGGUACCGGAAAGCAUATT
si*KIF26B*-2	CGGACAGCCUCUCCUAUUA
si*FOXD3*	GCAAUAGGGACGCGCCAAUTT

Supplementary Table 3. Primers for vectors construction

Primer Names	Sequences (5'-3')
pcDNA3.1(+)-*METTL3-FL*- Forward	CTTGGTACCGAGCTCGGATCCATGTCGGACACGTGGAGCTC
pcDNA3.1(+)-*METTL3-FL*- Reverse	TGCTGGATATCTGCAGAATTCGCTCTGTAAGGAAGTGCTTC
pcDNA3.1(+)-*FOXD3-FL* Forward	CTTGGTACCGAGCTCGGATCCATGACCCTCTCCGGCGGCGG
pcDNA3.1(+)-*FOXD3-FL* Reverse	AACGGGCCCTCTATACTCGAGCTATTGCGCCGGCCATTTGGCT
pcDNA3.1(+)/myc-His C-FOXD3-Forward	CTTGGTACCGAGCTCGGATCCATGACCCTCTCCGGCGGCGGCA
pcDNA3.1(+)/myc-His C-FOXD3-Reverse	TGCTGGATATCTGCAGAATTCTTGCGCCGGCCATTTGGCTT

pLKO.1-sh*KIF26B#1*-Forward	CCGGGGGACAACCGCTGTGACATTTTCTCGAGAGGGACAACCGCTGTGACATTTTTTTTTG
pLKO.1-sh*KIF26B#1*-Reverse	AATTCAAAAAAGGGACAACCGCTGTGACATTTTCTCGAGAGGGACAACCGCTGTGACATTT
pLKO.1-sh*KIF26B#2*-Forward	CCGGGCAACTCACACGTGTTCTTCATCTCGAGATGAAGAACACGTGTGAGTTGCTTTTTG
pLKO.1-sh*KIF26B#2*-Reverse	AATTCAAAAAAGCAACTCACACGTGTTCTTCATCTCGAGATGAAGAACACGTGTGAGTTGC
pLKO.1-sh*FOXD3#1*-Forward	CCGGGCCTAGTGAAGCCGCCTTACTTCTCGAGAAGTAAGGCGGCTTCACTAGGCTTTTTG
pLKO.1-sh*FOXD3#1*-Reverse	AATTCAAAAAGCCTAGTGAAGCCGCCTTACTTCTCGAGAAGTAAGGCGGCTTCACTAGGC
pLKO.1-sh*FOXD3#2*-Forward	CCGGATAGCTTTCCATACAGGTAAATCTCGAGATTTACCTGTATGGAAAGCTATTTTTG
pLKO.1-sh*FOXD3#2*-Reverse	AATTCAAAAAATAGCTTTCCATACAGGTAAATCTCGAGATTTACCTGTATGGAAAGCTAT

Supplementary Table 4. Antibodies

Antibodies		
anti-human m^{6}A polyclonal antibody (Dot Blot) (1:2000)	Synapic Systems	202003
anti-human m^{6}A polyclonal antibody (m^{6}A-RT-qPCR) (1:100)	ABclonal	A17924
anti-human METTL3 (1:1000)	ABclonal	A8370
anti-human METTL14 (1:1000)	ABclonal	A8530
anti-human FTO (1:1000)	Proteintech	27226-1-AP
anti-human ALKBH5 (1:1000)	Proteintech	16837-1-AP
anti-human YTHDF1 (1:1000)	Proteintech	17479-1-AP
anti-human YTHDF2 (1:1000)	Proteintech	24744-1-AP
anti-human YTHDF3 (1:1000)	Proteintech	25537-1-AP
anti-human YTHDC1 (1:1000)	Proteintech	14392-1-AP
anti-human YTHDC2 (1:1000)	Proteintech	27779-1-AP
anti-human P-YAP (Ser 127) (1:1000)	Cell Signaling Technology	13008

anti-human YAP (1:1000)	Proteintech	66900-1-Ig
anti-human P-MST1 (Thr183)/MST2(Thr180) (1:1000)	Cell Signaling Technology	49332
anti-human MST1 (1:1000)	Proteintech	22245-1-AP
anti-human P-LATS1 (Ser909) (1:1000)	Cell Signaling Technology	9157
anti-human LATS1 (1:1000)	Proteintech	17049-1-AP
anti-human FOXD3 (1:1000)	ABclonal	A2926
anti-human Lamin-B1 (1:1000)	Proteintech	12987-1-AP
anti-human KIF26B (1:500)	Proteintech	17422-1-AP
anti-human NF2 (1:1000)	ABclonal	A13626
anti-human KIBRA (1:1000)	ABclonal	A17110
anti-human FRMD6 (1:1000)	ABclonal	A9995
anti-human NF-κB p65 (1:1000)	Cell Signaling Technology	8242
anti-human Phospho-NF-κB p65 (1:1000)	Cell Signaling Technology	3033
anti-human Myc-Tag (1:100 for ChIP)	Cell Signaling Technology	2276S
Normal Rabbit IgG (5 μg for each IP sample)	Cell Signaling Technology	2729S
anti-human GAPDH (1:3000)	ABclonal	AC002
anti-human β-actin (1:3000)	ABclonal	AC026
HRP Goat Anti-Rabbit IgG (H+L) (1:3000)	ABclonal	AS014
HRP Goat Anti-Mouse IgG (H+L) (1:3000)	ABclonal	AS003
