# Supplementary material for: Fusobacterium nucleatum reduces METTL3-mediated m6A modification and contributes to colorectal cancer metastasis
Source: Nat Commun. 2022 Mar 10;13:1248. doi: 10.1038/s41467-022-28913-5 (PMC8913623; doi:10.1038/s41467-022-28913-5)
Supplement: Supplementary file 3 — Reporting Summary [file 41467_2022_28913_MOESM3_ESM.pdf]

nature research

Corresponding author(s): Wei Zhuo DBPR NCOMMS-21-01510B

Last updated by author(s): Feb 4, 2022

Reporting Summary

Nature Research wishes to improve the reproducibility of the work that we publish. This form provides structure for consistency and transparency in reporting. For further information on Nature Research policies, see our Editorial Policies and the Editorial Policy Checklist.

Statistics

For all statistical analyses, confirm that the following items are present in the figure legend, table legend, main text, or Methods section.

n/a	Confirmed	
☐	☒	The exact sample size (n) for each experimental group/condition, given as a discrete number and unit of measurement
☐	☒	A statement on whether measurements were taken from distinct samples or whether the same sample was measured repeatedly
☐	☒	The statistical test(s) used AND whether they are one- or two-sided<br>*Only common tests should be described solely by name; describe more complex techniques in the Methods section.*
☐	☒	A description of all covariates tested
☐	☒	A description of any assumptions or corrections, such as tests of normality and adjustment for multiple comparisons
☐	☒	A full description of the statistical parameters including central tendency (e.g. means) or other basic estimates (e.g. regression coefficient) AND variation (e.g. standard deviation) or associated estimates of uncertainty (e.g. confidence intervals)
☐	☒	For null hypothesis testing, the test statistic (e.g. F, t, r) with confidence intervals, effect sizes, degrees of freedom and P value noted<br>*Give P values as exact values whenever suitable.*
☒	☐	For Bayesian analysis, information on the choice of priors and Markov chain Monte Carlo settings
☒	☐	For hierarchical and complex designs, identification of the appropriate level for tests and full reporting of outcomes
☒	☐	Estimates of effect sizes (e.g. Cohen's d, Pearson's r), indicating how they were calculated

Our web collection on statistics for biologists contains articles on many of the points above.

Software and code

Policy information about availability of computer code

Data collection	For m6A sequencing, Illumina Hiseq platform was used to sequence the library. For RNA sequencing,150 bp Paired-end libraries were sequenced by Illumina PE150 platform. For imaging tumors in live animals, images were acquired with the Xenogen IVIS 200 Imaging System. For Immunofluorescence, fluorescent images were acquired using LSM 800 with Airyscan confocal laser-scanning microscope. Luciferase activity was measured with a dual luciferase reporter assay system (Promega).
Data analysis	For m6A sequencing, the significant peaks were analysis by MACS2. For RNA sequencing, the paired-end clean reads were aligned to the human genome version hg19 using Hisat2 v2.0.5. Differential expression analysis of two groups was performed using the DESeq2. Statistical analyses were performed using the GraphPad Prism 7 software. The dot blot and western blot results were quantified using Image J 1.53 software.

For manuscripts utilizing custom algorithms or software that are central to the research but not yet described in published literature, software must be made available to editors and reviewers. We strongly encourage code deposition in a community repository (e.g. GitHub). See the Nature Research guidelines for submitting code & software for further information.

Data

Policy information about availability of data

All manuscripts must include a data availability statement. This statement should provide the following information, where applicable:

- Accession codes, unique identifiers, or web links for publicly available datasets
- A list of figures that have associated raw data
- A description of any restrictions on data availability

Data availability statement
The accession number for the data for the m6A-seq reported in this study is NCBI GEO: GSE150308 . (Figure4A-E, Figure5A-B, Supplemental Figure 4A-D)

The accession number for the data for RNA-seq reported in this study is NCBI GEO: GSE150309 . (Figure6A-C)

Field-specific reporting

Please select the one below that is the best fit for your research. If you are not sure, read the appropriate sections before making your selection.

☒ Life sciences ☐ Behavioural & social sciences ☐ Ecological, evolutionary & environmental sciences

For a reference copy of the document with all sections, see nature.com/documents/nr-reporting-summary-flat.pdf

Life sciences study design

All studies must disclose on these points even when the disclosure is negative.

Sample size	For the animal assays, sample sizes and number of replicates were determined based on previous studies with models of colorectal cancer. For human samples, samples were collected until the sample size was sufficient to give reliable estimates. For cell and biochemical data, each experiment was performed at least three biological replicates.
Data exclusions	No data were excluded from the analyses.
Replication	Results were confirmed in at least three biological replicates for each experiment unless otherwise stated.
Randomization	All animals used were the same age and sex and were purchased from the same supplier, which were randomly divided into indicated groups.
Blinding	For the animal assays, the investigators were blinded to group allocation during injection.

Reporting for specific materials, systems and methods

We require information from authors about some types of materials, experimental systems and methods used in many studies. Here, indicate whether each material, system or method listed is relevant to your study. If you are not sure if a list item applies to your research, read the appropriate section before selecting a response.

Materials & experimental systems

n/a	Involved in the study	
☐	☒	Antibodies
☐	☒	Eukaryotic cell lines
☒	☐	Palaeontology and archaeology
☐	☒	Animals and other organisms
☐	☒	Human research participants
☒	☐	Clinical data
☒	☐	Dual use research of concern

Methods

n/a	Involved in the study	
☒	☐	ChIP-seq
☒	☐	Flow cytometry
☒	☐	MRI-based neuroimaging

Antibodies

Antibodies used	The antibodies used in the present study were: rabbit anti m6A (Synapic Systems, 202003, 1:2000 for Dot Blot), rabbit anti m6A (ABclonal, A17924, 1:100 for Me-Rip)rabbit anti METTL3 (ABclonal, A8370, 1:1000 for WB, 5 µg for each IP sample), rabbit anti METTL14 (ABclonal, A8530, 1:1000), rabbit anti FTO (Proteintech, 27226-1-AP, 1:1000), rabbit anti ALKBH5 (Proteintech, 16837-1-AP, 1:1000), rabbit anti YTHDF1 (Proteintech, 17479-1-AP, 1:1000), rabbit anti YTHDF2 (Proteintech, 24744-1-AP, 1:1000 for WB, 5 µg for each IP sample), rabbit anti YTHDF3 (Proteintech, 25537-1-AP, 1:1000), rabbit anti YTHDC1 (Proteintech, 14392-1-AP, 1:1000), rabbit anti YTHDC2 (Proteintech, 27779-1-AP, 1:1000), rabbit anti P-YAP (Ser 127) (Cell Signaling Technology, 13008, 1:1000), mouse anti YAP (Proteintech, 66900-1-Ig, 1:1000), rabbit anti P-MST1 (Thr183)/MST2(Thr180) (Cell Signaling Technology, 49332, 1:1000), rabbit anti MST1 (Proteintech, 22245-1-AP, 1:1000), rabbit anti P-LATS1 (Ser909) (Cell Signaling Technology, 9157, 1:1000), rabbit anti LATS1 (Proteintech, 17049-1-AP, 1:1000), rabbit anti FOXD3 (ABclonal, A2926, 1:1000), rabbit anti Lamin-B1 (Proteintech, 12987-1-AP, 1:1000), rabbit anti KIF26B (Proteintech, 17422-1-AP, 1:500), rabbit anti NF2 (ABclonal, A13626, 1:1000), rabbit anti KIBRA (ABclonal, A17110, 1:1000), rabbit anti FRMD6 (ABclonal, A9995, 1:1000), rabbit anti NF-κB p65 (Cell Signaling Technology, 8242, 1:1000), rabbit anti Phospho-NF-κB p65 (Cell Signaling Technology, 3033, 1:1000), mouse anti Myc-Tag (Cell Signaling Technology, 2276S, 1:100 for ChIP), Normal Rabbit IgG (Cell Signaling Technology, 2729S, 5 µg for each IP sample), mouse anti GAPDH (ABclonal, AC002, 1:3000), rabbit anti β-actin (ABclonal, AC026, 1:3000), HRP Goat Anti-Rabbit IgG (H+L) (ABclonal, S014, 1:3000), HRP Goat Anti-Mouse IgG (H+L) (ABclonal, AS003, 1:3000).
Validation	Molecular weight

Eukaryotic cell lines

Policy information about cell lines

Cell line source(s)	The CRC cell lines (HCT116, LoVo, RKO, SW620) were provided by the American Type Culture Collection (ATCC, Manassas, VA, USA).
Authentication	The lines were authenticated by short tandem repeat (STR) profiling.
Mycoplasma contamination	All cell lines used in this study were tested negative for mycoplasma contamination.
Commonly misidentified lines (See ICLAC register)	There is no commonly misidentified cell line used in this study.

Animals and other organisms

Policy information about studies involving animals; ARRIVE guidelines recommended for reporting animal research

Laboratory animals	Female BALB/c Nude mice (5-6 weeks old), NOD SCID mice (5-6 weeks old), C57BL/6 mice (5-6 weeks old), APC(Min/+) mice (2 month old) were used for indicated studies, kept in SPF facilities (25°C, suitable humidity (typically 50%), 12h dark/light cycle).
Wild animals	No wild animals were used.
Field-collected samples	No field-collected samples were used.
Ethics oversight	All mice used in this study were approved by the Institutional Animal Care and Use Committee of Zhejiang University.

Note that full information on the approval of the study protocol must also be provided in the manuscript.

Human research participants

Policy information about studies involving human research participants

Population characteristics	See above
Recruitment	All samples were obtained with informed consent from previously untreated CRC patients from Sir Run Run Shaw Hospital, Zhejiang University School of Medicine (Hangzhou, China). Ethical consent was approved by the Institutional Review Board of Sir Run Run Shaw Hospital.
Ethics oversight	All samples were obtained with informed consent from previously untreated CRC patients from Sir Run Run Shaw Hospital, Zhejiang University School of Medicine (Hangzhou, China). Ethical consent was approved by the Institutional Review Board of Sir Run Run Shaw Hospital.

Note that full information on the approval of the study protocol must also be provided in the manuscript.
